# A Case of Congenital Syphilis Presenting with Unusual Skin Eruptions

**DOI:** 10.1155/2018/1761454

**Published:** 2018-03-25

**Authors:** Alexander K. C. Leung, Kin Fon Leong, Joseph M. Lam

**Affiliations:** ^1^Department of Pediatrics, Alberta Children's Hospital, University of Calgary, Calgary, AB, Canada T2M 0H5; ^2^Pediatric Institute, Kuala Lumpur General Hospital, Kuala Lumpur, Malaysia; ^3^Department of Pediatrics, Department of Dermatology and Skin Sciences, University of British Columbia, Vancouver, BC, Canada V5Z 1K1

## Abstract

Once believed to be a rare disease in developed countries, recent data suggest that there is a surge in incidence of congenital syphilis in many developed countries. Diagnosis of congenital syphilis can be difficult because more than two-thirds of affected infants are asymptomatic at birth, and signs of symptomatic infants may be nonspecific or subtle. On top of this, some affected infants may have atypical presentations. Familiarity with the diverse presentations is essential to diagnosis. We report a 2-week-old male infant with congenital syphilis whose cutaneous manifestations included diffuse, erythematous keratoderma with desquamation and fissures on his hands and feet, multiple linear scaly fissures at the angles of his mouth, and onychauxis of the fingernails and toenails To our knowledge, diffuse, erythematous keratoderma of the hands and feet and thick nails have not been reported previously in congenital syphilis.

## 1. Introduction

Congenital syphilis is an infectious disease resulting mainly from hematogenous transmission of *Treponema pallidum*, a spirochete bacterium, through the placenta of an infected mother to the fetus during pregnancy [1]. Occasionally, it can be acquired through direct contact with infectious lesions in the birth canal or on the perineum of the mother during the birth process [2–4]. Diagnosis of congenital syphilis can be difficult because more than two-thirds of affected infants are asymptomatic at birth, and signs of symptomatic infants may be nonspecific or subtle [1, 2, 5]. On top of this, some affected infants may have atypical presentations. Familiarity with the diverse presentations is essential to early diagnosis. We report a 2-week-old male infant with congenital syphilis whose cutaneous manifestations included diffuse, erythematous keratoderma with desquamation and fissures on his hands and feet, multiple linear scaly fissures at the angles of his mouth, and long and thick fingernails and toenails. To the best of our knowledge, diffuse, erythematous keratoderma of the hands and feet and onychauxis of the fingernails and toenails have not been reported previously in congenital syphilis.

## 2. Case Report

A 2-week-old male infant presented with asymptomatic, erythematous, desquamative lesions on the hands and feet which were noted at birth. He was born to a 30-year-old, G2P2 mother who had poor prenatal care for social and economic reasons. The mother did not have a venereal disease research laboratory (VDRL) test or rapid plasma reagin (RPR) test during the pregnancy. Parents were sexually active and had multiple sexual partners. They were nonconsanguineous, asymptomatic, and apparently healthy. The infant was born at 38 weeks gestation following a normal spontaneous vaginal delivery. Apgar scores were 5 and 7 at 1 and 5 minutes, respectively. Birth weight was 2.8 kg, length 45.8 cm, and head circumference 33 cm. The infant was formula fed. He had a poor appetite and was not thriving.

**Figure 1:**
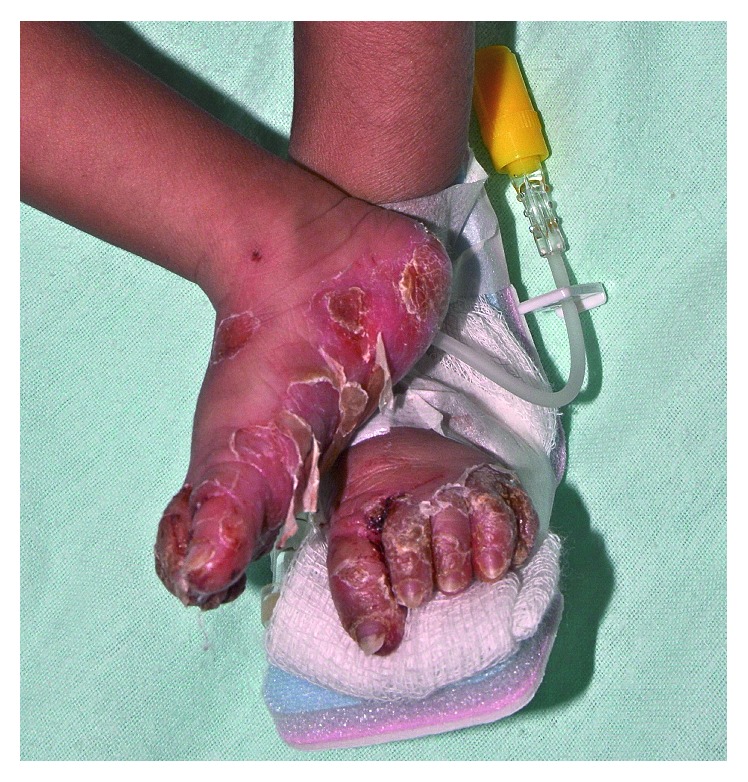
Diffuse, erythematous, transgradient palmoplantar keratoderma with desquamation and fissures.

**Figure 2:**
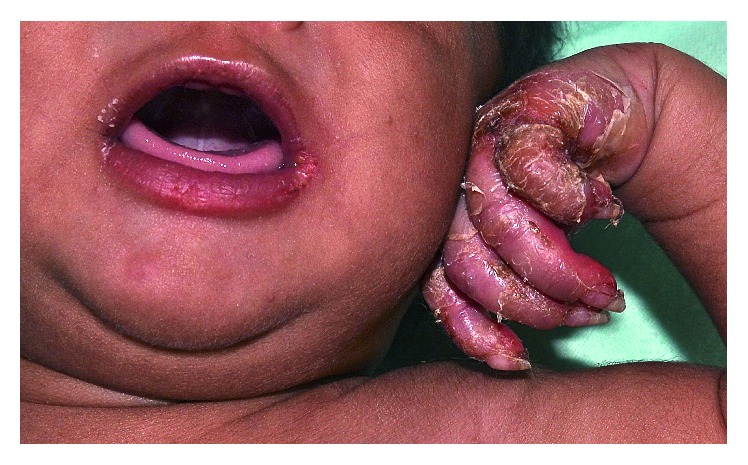
Multiple linear scaly fissures noted at the angles of the mouth.

On examination, his weight was 2.85 kg (3rd percentile), length 46 cm (1 cm below 3rd percentile), and head circumference 33.2 cm (1 cm below 3rd percentile). Vital signs were stable (temperature 37.2°C, heart rate 82 beats per minute, and respiratory rate 30 breaths per minute). The infant was pale and mildly irritable. There were no dysmorphic features. He had a normal muscle tone and no focal neurological deficits. Movements of the extremities were normal. He had nasal congestion and snuffles. Skin examination revealed diffuse, erythematous, palmoplantar keratoderma with desquamation and fissures on his hands and feet, especially over the palms and soles (Figures 1 and 2). Multiple linear scaly fissures were noted at the angles of his mouth (Figure 2). The fingernails and toenails were thick and long (Figures 1 and 2). The abdomen was slightly distended. The liver was 2 cm below the costal margin, and the spleen tip was palpable. There was no lymphadenopathy. The ophthalmologic examination was normal. The rest of the examination was unremarkable. In particular, there was no mucous membrane involvement or hair abnormality.

A complete blood cell count demonstrated a hemoglobin of 9.3 g/dL, reticulocyte count of 6%, white cell count of 7,250/*µ*l, and platelet count of 97,000/*µ*L. Coombs test was negative. His serum total protein was 5 g/dL with an albumin level of 2.8 g/dL. C-reactive protein (CRP) was 7 mg/L. Results of liver function tests were normal with an aspartate aminotransferase (AST) level of 27 IU/L, alanine aminotransferase (ALT) level 14 IU/L, and total bilirubin level of 0.3 mg/dL. Serologic tests for toxoplasmosis, rubella, cytomegalovirus, Epstein–Barr virus, and herpes simplex virus were all negative. The infant had a positive VDRL titer of 1 : 256 and a positive *Treponema pallidum* hemagglutination (TPHA) test. Concurrently, the mother had a positive VDRL titer of 1 : 16 and a positive TPHA test. Given these findings, a diagnosis of congenital syphilis was made. The infant's cerebrospinal fluid (CSF) examination was normal, and CSF-VDRL test was negative. No microorganism was identified in culture specimens of blood, urine, or CSF. Radiographs of long bones were normal. Hearing test was normal.

**Figure 3:**
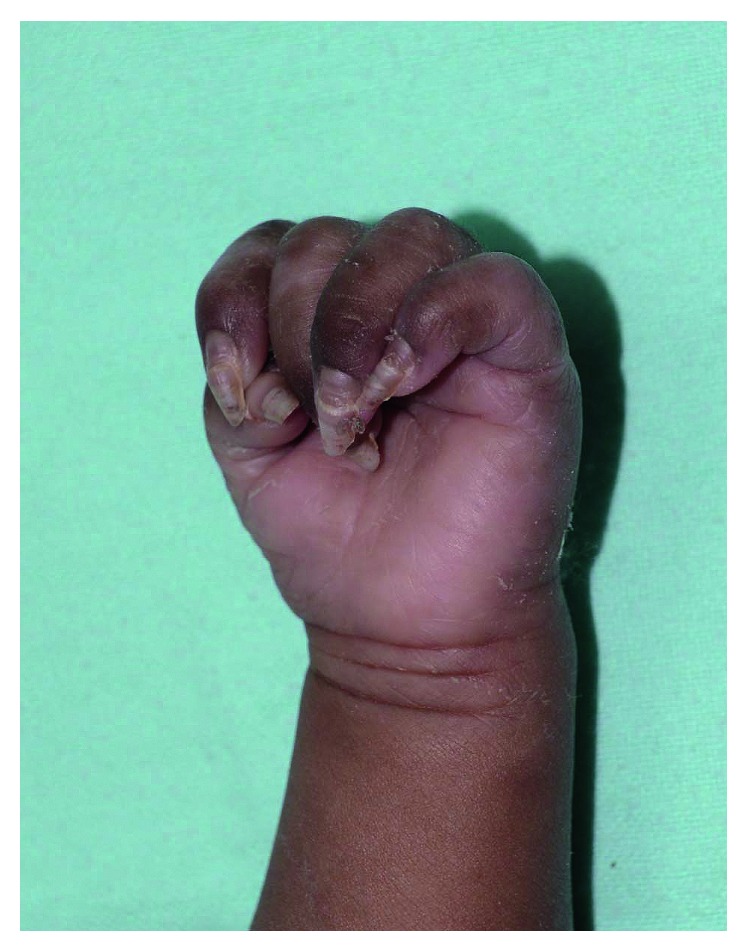
Resolution of palmoplantar keratoderma with residual onychauxis after 14 days of treatment.

The patient was treated with intravenous aqueous crystalline penicillin 50,000 u/kg/dose every 8 hours for a total of 10 days. His skin eruption, except the thick nails, resolved after 14 days of treatment (Figure 3). Contact tracing was initiated, and his parents were treated with procaine penicillin 2.4 million units intramuscularly as a single dose. At one month of age, the infant had a hemoglobin of 10.5 g/dL, reticulocyte count of 2%, white cell count of 7,630/*µ*l, and platelet count 235,000/*µ*L. The patient was followed up with VDRL titer, physical examination, and measurement of growth parameters. The VDRL titer decreased to 1 : 2 dilutions at 3 months. At 6-month follow-up, physical examination, including examination of the nails, was normal. The infant was thriving well with a weight of 7.4 kg (between the 10th and 25th percentiles) and length of 65 cm (10th percentile).

## 3. Discussion

Congenital syphilis is a serious public health issue. Once believed to be a rare disease in developed countries, recent data suggest that there is a surge in incidence of congenital syphilis in North America and Europe [1, 6, 7]. In the United States, the reported rate of congenital syphilis was 100 cases per 100,000 live births in 1991, reached a historical nadir of 8.2 cases per 100,000 live births in 2005, and surged to 11.6 cases per 100,000 live births in 2014 [1, 6, 7]. Congenital syphilis can lead to spontaneous abortion, intrauterine growth retardation, nonimmune hydrops fetalis, stillbirth, prematurity, and perinatal death as well as severe sequelae and even mortality in some live-born infants [1, 3, 8].

Congenital syphilis is arbitrarily divided into early and late congenital syphilis. The former has onset of clinical manifestations before 2 years of age while the latter has onset of clinical manifestations after 2 years of age (usually manifesting around puberty) [5, 7, 9]. Although the majority of infants with congenital syphilis are asymptomatic at birth, among symptomatic ones, clinical manifestations of early congenital syphilis include skin rash, snuffles, jaundice, hepatomegaly with or without splenomegaly, fever, generalized lymphadenopathy, and failure to thrive [5, 7, 8]. Affected infants may have Coombs-negative hemolytic anemia, thrombocytopenia, neurosyphilis, pneumonia, hepatitis, and skeletal abnormalities [8, 10]. Skeletal abnormalities may be in the forms of lucencies (demineralization) and erosions (osseous destruction) of the proximal medial tibial metaphysis (Wimberger sign), metaphyseal lucent bands, metaphyseal serrated appearance at the epiphyseal margin of long bones (Wegner sign), diaphyseal periostitis, irregular areas of increased density and rarefaction (“moth-eaten” appearance), and multiple sites of osteochondritis [1, 7, 11]. The osteochondritis is painful, resulting in the refusal to move the involved limb (pseudoparalysis of Parrot) [5, 7, 8].

Clinical manifestations of late congenital syphilis include perioral fissures (rhagades), saddle nose deformity, frontal bossing, Hutchinson's triad (peg-shaped, notched, widely spaced permanent upper central incisors; interstitial keratitis; and the eighth cranial nerve deafness), multicusped first molars (mulberry molars), mental retardation, perforation of the hard palate, prognathism, painless effusion of knees (Clutton joints), thickening of sternoclavicular joint (Higoumenakis sign), scaphoid scapula, and anterior bowing of shins (saber shins) [5, 7, 9, 12].

The most common cutaneous finding of early congenital syphilis is a symmetrical, copper-red maculopapular rash [1, 7, 10] Less commonly, the eruption may be in the form of acral skin desquamation, acral vesiculobullae (pemphigus syphiliticus), mucous patches, petechiae, erythema multiforme-like targetoid lesions, perioral/perinasal/perianal fissures, and condylomata lata [1, 4, 6, 7, 10, 13]. Generally, skin lesions are often the clue that leads to clinical suspicion of congenital syphilis.

Our patient had congenital syphilis as suggested by the infant's positive VDRL titer of 1 : 256 which was more than four times of the concurrent mother's titer of 1 : 16 [3]. The diagnosis was confirmed by a positive TPHA test. Our patient had clinical manifestations of early congenital syphilis such as acral skin desquamation, perioral fissures, snuffles, hepatomegaly, failure to thrive, Coombs-negative hemolytic anemia, and thrombocytopenia. In addition, he also had diffuse, erythematous keratoderma of the hands and feet and thick fingernails and toenails. The latter two features have not been reported previously in patients with congenital syphilis. We suggest adding palmoplantar keratoderma and onychauxis to the list of cutaneous manifestations of early congenital syphilis.

## 4. Conclusion

A once forgotten disease is now back. Congenital syphilis is not gone but often forgotten. We report a 2-week-old infant who presented with unusual manifestations of congenital syphilis. Physicians should be aware of the diverse clinical features of congenital syphilis and have a high index of suspicion so that a correct diagnosis can be made and treatment can be initiated early.
